# Genome Sequence of *Desulfurella amilsii* Strain TR1 and Comparative Genomics of *Desulfurellaceae* Family

**DOI:** 10.3389/fmicb.2017.00222

**Published:** 2017-02-20

**Authors:** Anna P. Florentino, Alfons J. M. Stams, Irene Sánchez-Andrea

**Affiliations:** ^1^Laboratory of Microbiology, Wageningen UniversityWageningen, Netherlands; ^2^Sub-department of Environmental Technology, Wageningen UniversityWageningen, Netherlands; ^3^Centre of Biological Engineering, University of MinhoBraga, Portugal

**Keywords:** comparative genomics, *Desulfurellaceae*, sulfur reducers, acidophiles, metabolism

## Abstract

The acidotolerant sulfur reducer *Desulfurella amilsii* was isolated from sediments of Tinto River, an extremely acidic environment. Its ability to grow in a broad range of pH and to tolerate certain heavy metals offers potential for metal recovery processes. Here we report its high-quality draft genome sequence and compare it to the available genome sequences of other members of *Desulfurellaceae* family: *D. acetivorans. D. multipotens, Hippea maritima. H. alviniae, H. medeae*, and *H. jasoniae*. For most species, pairwise comparisons for average nucleotide identity (ANI) and *in silico* DNA–DNA hybridization (DDH) revealed ANI values from 67.5 to 80% and DDH values from 12.9 to 24.2%. *D. acetivorans* and *D. multipotens*, however, surpassed the estimated thresholds of species definition for both DDH (98.6%) and ANI (88.1%). Therefore, they should be merged to a single species. Comparative analysis of *Desulfurellaceae* genomes revealed different gene content for sulfur respiration between *Desulfurella* and *Hippea* species. Sulfur reductase is only encoded in *D. amilsii*, in which it is suggested to play a role in sulfur respiration, especially at low pH. Polysulfide reductase is only encoded in *Hippea* species; it is likely that this genus uses polysulfide as electron acceptor. Genes encoding thiosulfate reductase are present in all the genomes, but dissimilatory sulfite reductase is only present in *Desulfurella* species. Thus, thiosulfate respiration via sulfite is only likely in this genus. Although sulfur disproportionation occurs in *Desulfurella* species, the molecular mechanism behind this process is not yet understood, hampering a genome prediction. The metabolism of acetate in *Desulfurella* species can occur via the acetyl-CoA synthetase or via acetate kinase in combination with phosphate acetyltransferase, while in *Hippea* species, it might occur via the acetate kinase. Large differences in gene sets involved in resistance to acidic conditions were not detected among the genomes. Therefore, the regulation of those genes, or a mechanism not yet known, might be responsible for the unique ability of *D. amilsii*. This is the first report on comparative genomics of sulfur-reducing bacteria, which is valuable to give insight into this poorly understood metabolism, but of great potential for biotechnological purposes and of environmental significance.

## Introduction

Elemental sulfur reduction is a respiratory-chain dependent redox process that yields ATP by utilizing sulfur as an oxidizing agent. This metabolism is of great importance for the biogeochemical cycle of sulfur in extreme environments, from where sulfur reducers have most frequently been isolated (Bonch-Osmolovskaya et al., 1990; Stetter, 1996; Alain et al., 2009; Birrien et al., 2011). Sulfur reduction leads to the formation of sulfide, a compound that, despite its corrosive properties, has an important role in biotechnological applications, such as metal precipitation (Johnson and Hallberg, 2005). Early assumptions considered sulfur reduction to be of low physiological importance as reviewed by Rabus et al. (2006). However, it is now known that this metabolism can yield energy for growth coupled to the utilization of several electron donors, such as alcohols, organic acids, and sugars (Bonch-Osmolovskaya et al., 1990; Finster et al., 1997; Dirmeier et al., 1998; Boyd et al., 2007; Florentino et al., 2016b); and the majority of sulfur-reducing microorganisms are able to grow chemolithotrophically (Segerer et al., 1986; Bonch-Osmolovskaya et al., 1990; Caccavo et al., 1994; Stetter, 1996; Miroshnichenko et al., 1999). Although sulfur-reducing microorganisms have a versatile metabolism (Dirmeier et al., 1998; Boyd et al., 2007), little attention has been paid to its genomic features beyond the biochemistry and bioenergetics of the process.

From current observations, microorganisms able to reduce elemental sulfur are spread over more than a 100 genera in the tree of life (Florentino et al., 2016a). In the *Bacteria* domain, the majority of the sulfur-reducing species align within the *Proteobacteria* phylum. In this group, the *Desulfurellaceae* family comprises the genera *Desulfurella* and *Hippea*, inhabiting terrestrial environments and submarine hot vents, respectively (Blumentals et al., 1990; Greene, 2014). Although the genomes of several members of the *Desulfurellaceae* family are sequenced, *Hippea maritima* is the only species with its genome description reported.

*Desulfurella amilsii*, an acidotolerant sulfur reducer, was recently isolated from sediments of the Tinto River, an extreme acidic environment (Florentino et al., 2015). The phenotypic characterization of *D. amilsii* revealed its ability to utilize not only sulfur but also thiosulfate as an electron acceptor (as was reported for *D. propionica*) and to ferment pyruvate (as also reported for *D. acetivorans*). Unlike other members in the *Desulfurellaceae* family, *D. amilsii* utilizes formate as an electron donor and thrives at pH as low as 3 (Florentino et al., 2016a). The utilization of acetate is common among the species. However, the ability of *D. amilsii* to metabolize it at low pH is peculiar, since at acidic conditions, acetate is protonated and become acetic acid, a toxic compound for most prokaryotic species (Holyoak et al., 1996).

The respiration of elemental sulfur is thought to be coupled to ADP phosphorylation, in which hydrogenases or dehydrogenases transfer electrons to sulfur-reducing enzymes via electron carriers, such as menaquinones or cytochromes (Rabus et al., 2006) together with proton translocation. The biochemical mechanisms via which microorganisms reduce elemental sulfur to H_2_S and the nature of the enzymes involved in the process are not yet completely understood, especially at low pH. The low solubility of elemental sulfur in aqueous medium (5 μg L^-1^ at 20°C) and the chemical transformation of sulfur compounds, that is dependent on pH, hamper a broad understanding of sulfidogenic processes (Schauder and Müller, 1993; Florentino et al., 2016b). Some microorganisms, as for example *Wolinella succinogenes* (Macy et al., 1986) can overcome the low solubility of elemental sulfur by utilizing more hydrophilic forms of the compound, such as polysulfides. In aqueous solution containing nucleophiles, such as sulfide or cysteine, elemental sulfur can be readily converted to polysulfide (Blumentals et al., 1990; Schauder and Müller, 1993), particularly at neutral and high pH levels. The most studied sulfur reducers are neutrophiles where the enzymes that have been suggested to use polysulfide as a substrate -sulfhydrogenase (SH) and polysulfide reductase (PSR) – are targeted (Macy et al., 1986). However, the instability of polysulfide at low pH, makes it an unlikely substrate for acidophiles.

A sulfur reductase (SRE) was purified from the membrane fraction of *Acidianus ambivalens*, which respires elemental sulfur in a range of pH from 1 to 3.5 (Laska et al., 2003). This enzyme uses elemental sulfur as a substrate and seems to be responsible for sulfur respiration at low pH values, where the formation of soluble intermediates, such as polysulfide is unlikely. Therefore, direct contact is hypothesized to be essential for elemental sulfur reduction at low pH (Stetter and Gaag, 1983; Pihl et al., 1989; Finster et al., 1998; Laska et al., 2003). The mechanisms by which sulfur reducers get access to insoluble sulfur, however, are still unclear.

Although the optimum pH for growth of *Desulfurellaceae* members is approximately neutral (6.0–7.0), *D. acetivorans* withstands pH as low as 4.3 for its growth. However, the ability of *D. amilsii* to thrive at very acidic conditions, pH as low as 3, is unique in the *Desulfurellaceae* family, which makes it a potential catalyst for biotechnological processes, such as metal precipitation from acidic waste streams. To get insights into the encoded pathways for sulfur reduction by this strain, we analyzed the genome of *D. amilsii* and compared it with available genome sequences of other members within the *Desulfurellaceae* family. To the best of our knowledge, there is no reported study on comparative genomics of acidophilic sulfur-reducing microorganisms adapted to different conditions.

## Materials and Methods

### Cultivation, Genome Sequencing and Assembly

For genome sequencing, a 500-mL culture of *D. amilsii* was grown on acetate and sulfur as described elsewhere (Florentino et al., 2015). Cells were harvested at the early stationary phase, when the sulfide production in the culture reached 10 mM, by centrifuging at 19000 × *g* for 20 min. Genomic DNA was extracted using the MasterPure^TM^ Gram Positive DNA Purification Kit (Epicentre, Madison, WI, USA), following the instructions of the manufacturer. The genome was sequenced using the Illumina HiSeq2000 paired-end sequencing platform of GATC Biotech (Konstanz, Germany). Sequence assembly was performed using two independent assemblers: the OLC-assembler Edena (Hernandez et al., 2008) and the de-Bruijn-Graph-assembler Ray (Boisvert et al., 2010). Sets of overlapping sequences were identified from both assembling procedures and further merged into a more contiguous and consistent assembly, using the hybrid sequencing technology assembler Zorro (Argueso et al., 2009). The obtained sequences were further improved by scaffolding with Opera and by gap-closing with GapFiller (Boetzer and Pirovano, 2012). The closed gaps were manually verified.

### Genome Annotation

Automated annotation was performed using the RAST annotation server (Aziz et al., 2008), followed by manual curation. Additional gene prediction analysis and functional annotation were done within the Integrated Microbial Genomes – Expert Review from the DOE – Joint Genome Institute pipeline (Markowitz et al., 2014). The predicted coding sequences (CDSs) were translated into amino acid sequences and used in homology searches in the National Center for Biotechnology Information (NCBI) non-redundant database and the Uniprot, TIGRFam, Pfam, SMART, PRIAM, KEGG, COG, and Interpro databases. These data sources were combined to assign a product description for each predicted protein. Clusters of regularly interspaced repeats (CRISPR) were identified via the web available tools CRISPRFinder (Grissa et al., 2007) and CRISPRTarget (Biswas et al., 2013). The N-terminal twin arginine translocation (Tat) signal peptides and the transmembrane helices were predicted using the online tools from TMHMM server v. 2.03^1^ and PROTTER v. 1.0^2^.

The Whole Genome Shotgun project of *Desulfurella amilsii* has been deposited at DDBJ/ENA/GenBank under the accession MDSU00000000. The version described in this paper is version MDSU01000000. The genome ID in the integrated microbial genomes-expert review (IMG) database is 2693429826.

### Comparative Genomics

The genome sequences used for the comparative study (and their accession numbers) were: *D. acetivorans* strain A63 (CP007051), *D. multipotens* strain RH-8 (SAMN05660835), *H. maritima* strain MH2 (CP002606), *H. alviniae* strain EP5-r (ATUV00000000), *H. medeae* strain KM1 (JAFP00000000), and *H. jasoniae* strain Mar08-272r (JQLX00000000).

The average nucleotide identity analysis (ANI) between the genome dataset pairs was performed using the online tool ANI calculator, available at http://enve-omics.ce.gatech.edu/ani/index. The best hits (one-way ANI) and the reciprocal best hits (two-way ANI) were considered, as calculated by Goris et al. (2007). *In silico* DNA–DNA hybridization (DDH) values were determined using the recommended settings of the Genome-to-Genome Distance Calculator (GGDC) web server version 2.0 (Meier-Kolthoff et al., 2013).

The number of genes shared between *Desulfurella* and *Hippea* species was assessed by OrthoMCL tool (Wang et al., 2015) and a Venn diagram was built using the web-based tool InteractiVenn (Heberle et al., 2015). Orthology between two genes was defined as best bidirectional hits, which were required to have at least 30% identity over at least 80% coverage of both sequences (Chen et al., 2006). All analyzed genes and predicted proteins from the *Desulfurellaceae* members’ genomes were compared using BLAST (Altschul et al., 1990).

The genomes were compared in terms of gene content using the ‘Phylogenetic Profiler for Single Genes’ of JGI-IMG website^3^ to identify genes in the query genome that have homologs present or absent in other genomes. The ‘Phylogenetic Profiler for Gene Cassettes’ tool of the same website was also used to find part of a gene cassette in a query genome, as well as conserved part of gene cassettes in other genomes. In terms of functional capabilities, comparisons of relative abundance of protein families (COGs, Pfams, TIGRfams) across selected genomes were performed with the ‘Abundance Profile Overview’ and ‘Function Profile’ tools. The potential metabolic capabilities of genomes were compared in the context of KEGG pathways.

## Results and Discussion

### General Characteristics of the *D. amilsii* Genome

The *D. amilsii* genome consists of 2.010.635 bp with a G + C content of 33.98% mol/mol. The initial sequencing resulted in 2.287.922 paired-end reads with a length of 301 bases, which were assembled into 20 contigs with a 687 fold coverage and a completeness of 99.9%. The largest scaffold consisted of 1,269,579 bp and the second and third largest scaffolds together consisted of 400,000 bp, covering more than 85% of the genome.

From the 2137 genes predicted by automated annotation in the genome, 49 were tRNA and rRNA genes, and 2088 protein coding genes (CDS). Two identical copies of the 16S rRNA gene (100% similarity) were identified. From the 2088 CDS (**Supplementary Table S1**), 1625 were predicted to have assigned COGs function, whilst 680 could not be assigned to any function in the database, and therefore were annotated as hypothetical proteins or proteins of unknown function. No pseudo genes were detected in the genome of *D. amilsii*, which is a unique characteristic in the *Desulfurellaceae* family. Two CRISPR regions were identified in the genome of 684 bp length with 10 spacers, and 291 bp length with 4 spacers, respectively. The spacers’ sequences from the first locus match viral DNA sequences found in several species in a BLAST based search, including *Bacillus* sp., *Ralstonia* sp., *Shewanella* sp., *Acinetobacter* sp., *Propionibacterium* sp., *Campylobacter* sp., *Escherichia* sp., *Staphylococcus* sp., *Sphingomonas* sp., and *Moraxella* sp. The spacer’s sequences related to the second locus match sequences of viral DNA also detected in *Edwardsiella hoshinae. Owenweeksia hongkongensis. Parascaris equorum*, and *Ovis canadensis* species.

The genome encodes a complete tricarboxylic acid (TCA) cycle pathway (**Supplementary Table S2**). Besides, routes for pyruvate fermentation are encoded, and physiological tests revealed acetate, hydrogen and CO_2_ as the end products (Florentino et al., 2016a). *D. amilsii* is able to grow chemolithotrophically; the CO_2_ fixation could be possible via the reductive TCA cycle for which all the genes are encoded (**Supplementary Table S3**). The genome encodes Ni–Fe, Ni–Fe–Se, and Fe–S hydrogenases, an intracellular formate dehydrogenase (FDH) and a formate-hydrogen lyase. Genes encoding for dinitrogenase iron-molybdenum cofactor, nitrogen fixation protein NifU and glutamine synthetase type I are present in the genome and might be involved in nitrogen fixation by *D. amilsii*. Sulfur and thiosulfate were reported to serve as electron acceptors for this microorganism (Florentino et al., 2015, 2016a) and genes essential for sulfur and thiosulfate reduction are encoded (**Supplementary Table S3**). Moreover, the importance of electron transport in this microorganism is highlighted by a high number of electron transport related genes (159). Genes encoding resistance to acidic conditions (**Supplementary Table S4**), oxygen stress tolerance (**Supplementary Table S5**), and metals resistance (**Supplementary Table S6**) are also identified, which is in line with the reported ability of the microorganism to grow at pH as low as 3 (Florentino et al., 2016a) and in the presence of heavy metals in solution (Florentino et al., 2015).

### Comparative Genomics

#### ANI and *In silico* DDH Analysis

Average nucleotide identity and *in silico* DDH values obtained from pairwise comparison of the available genome sequences of *Desulfurellaceae* family members are shown in **Table 1**. ANI values in the range of ≥95–96% correspond to ≥70% DDH standard for species definition (Goris et al., 2007). In general, the values are consistent with their phylogenetic relationships. While the taxonomic status of *D. amilsii* is well supported by the genomic signatures analysis, ANI and DDH values of *D. multipotens* and *D. acetivorans* were 98.6 and 88.1% respectively, surpassing the thresholds for species definition. The wet laboratory DNA–DNA hybridization experiment reported a borderline value of 69 ± 2% (Miroshnichenko et al., 1994) and the phylogenetic reconstruction of the *Desulfurella* genus shown by Florentino et al. (2016a) revealed more than 99.9% shared identity of 16S rRNA sequences for the two strains, while all the other members of the *Desulfurellaceae* family shared 92.1–97.7% identity (**Supplementary Table S7**).

**Table 1:** Average nucleotide identity and *in silico* DNA–DNA hybridization pairwise comparison of the available genomes sequences of *Desulfurellaceae* family.

	Average nucleotide identity (ANI)						
	
	*Dam*	*Dac*	*Dmu*	*Hma*	*Hme*	*Hal*	*Hja*
1		80.0	80.0	68.4	67.5	68.7 (±0.1)	68.4
2	21.9 (±2.4)		**98.6**	68.9 (±0.1)	69.1 (±0.2)	69.8	69.1
3	21.8 (±2.4)	**88.1 (±2.3)**		68.8	67.8	69.4	69.0
4	24.2 (±2.4)	23.7 (±2.4)	23.2 (±2.4)		78.7	74.0 (±0.1)	72.9
5	27.2 (±2.4)	23.9 (±2.4)	24.1 (±2.4)	20.7 (±2.3)		73.4	72.6
6	21.6 (±2.4)	17.4 (±2.2)	17.0 (±2.2)	16.9 (±2.2)	12.9 (±2.5)		73.1
7	16.5 (±6.4)	14.9 (±3.5)	14.9 (±3.5)	16.1 (±1.0)	15.8 (±1.4)	16.3 (±0.7)	

	**DNA–DNA hybridization (DDH)**						


*Dam, *D. amilsii*; Dac, *D. acetivorans*; Dmu, *D. multipotens*; Hma, *H. Maritima;* Hme, *H. medeae*; Hal, *H. alviniae*; Hja, *H. jasoniae*. The table is split by the empty diagonal cells; the ANI values are shown on the upper side and the *in silico* DDH values are shown on the lower side. Standard deviation values derived from bi-directional calculation are shown in brackets when they differed from 0. Values in bold highlight the off-limit comparison between *D. acetivorans* and *D. multipotens*.*

The physiological characterization of these two strains revealed different abilities to utilize butyrate and H_2_ as electron donors, which are oxidized by *D. multipotens* (Miroshnichenko et al., 1994) but not by *D. acetivorans* (Bonch-Osmolovskaya et al., 1990). Furthermore, the generation time was shown to be 2 h for *D. acetivorans*, while it was 5 h for *D. multipotens*, although generation time can generally vary with the growth conditions. The optimum range of temperature for growth ranged from 52–55°C in *D. acetivorans* (Bonch-Osmolovskaya et al., 1990) to 58–60°C in *D. multipotens* (Miroshnichenko et al., 1994). No chemotaxonomic information is provided in the characterization manuscripts of the mentioned strains. Although the characterization studies showed a G + C content of 31.4% mol/mol for *D. acetivorans* (Bonch-Osmolovskaya et al., 1990) and 33.5% mol/mol for *D. multipotens* (Miroshnichenko et al., 1994), the G + C content calculation based on the genome sequences shows no difference between them, with 32% mol/mol of G + C content. Despite the mentioned different physiological characteristics mentioned, the ANI values combined with an *in silico* DDH evaluation and a phylogenetic analysis of the 16S rRNA sequences support the similarity of both strains. Therefore, *D. multipotens* and *D. acetivorans* might belong to the same species and should be reclassified. Due to this finding, the comparative genomics described in this manuscript was performed with *D. acetivorans* as representative of *D. multipotens*, as it was the first species described and so represents the type strain of the genus.

**FIGURE 1:**
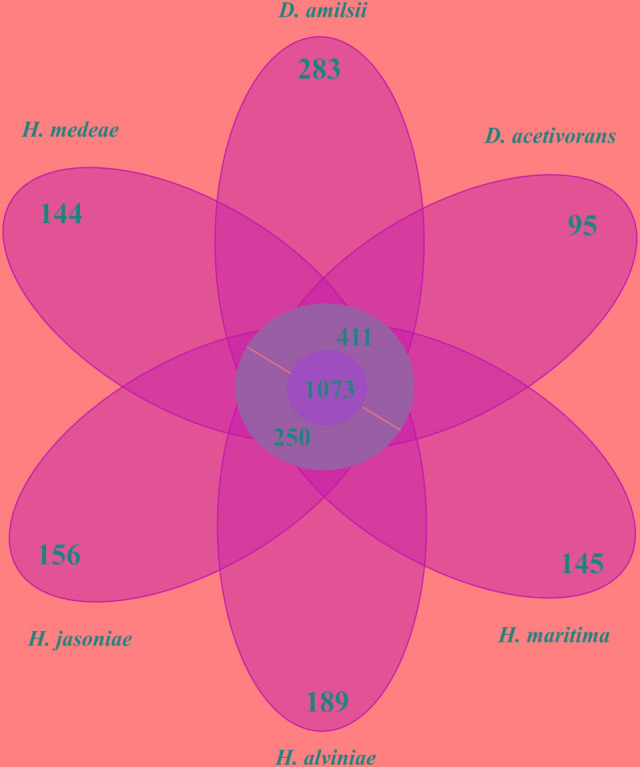
**Venn-diagram of the orthologous clusters of genes for *Desulfurellaceae* family members**.

In general, members of the *Desulfurellaceae* family possess a small genome, ranging from 1.7 to 2.0 Mbp of which more than 93% represent DNA coding regions, 80% of proteins with a predicted function and 70% of clusters of orthologous groups of proteins (COGs). General features of the genomes are compared in **Table 2**. In total, 2738 clusters of orthologous groups with functional prediction were found within the six members studied as shown in a Venn-diagram (**Figure 1**). The core genome consisted of 1073 shared sequences, 411 sequences shared by both *Desulfurella* genomes and 250 shared within the *Hippea* genus. *D. amilsii* showed the biggest genome size in the family and the biggest number of unique genes encoded, 283 (**Supplementary Table S8**), from which 62% are related to hypothetical proteins. Divergences in unique and shared gene sets might also explain other differences that have been found when conducting comparative studies on metabolism among the species, especially with respect to enzymes involved in sulfur reduction, sulfur disproportionation, pyruvate fermentation, and formate utilization.

**Table 2:** General genome features of *Desulfurellaceae* members.

Features	*D. amilsii*	*D. acetivorans*	*H. maritima*	*H. alviniae*	*H. medeae*	*H. jasoniae*
Strain	TR1	A63	MH2	EP5-r	KM1	Mar08-272r
DSM number	29984	5264	10411	24586	–	24585
Genome size (Mbp)	2.0	1.8	1.7	1.7	1.7	1.7
Completeness (%)	99.9	100	99.1	72.6	99.1	100
DNA coding	1877485	1731246	1624527	1672554	1669463	1655666
G+C (%)	33.98	32.08	37.47	37.03	42.85	37.00
Scaffolds	20	2	1	4	1	18
Total genes	2135	1875	1780	1814	1776	1768
CDS	2086	1819	1723	1757	1719	1710
RNA genes	49	56	57	57	57	58
tRNA genes	45	48	48	46	48	46
Pseudo genes	–	53	46	39	23	11
Function prediction	1723	1586	1498	1477	1499	1495
COGs	1456	1402	1287	1327	1320	1306
Pfam domains	1719	1633	1529	1535	1541	1536
CRISPR counts	2	3	–	1	4	–


### Sulfur Reduction and Energy Conservation

The electron transport chain in sulfur reducers normally links hydrogenases or dehydrogenases to membrane bound or cytoplasmic sulfur/polysulfide reductases (Laska et al., 2003; Fauque and Barton, 2012; Florentino et al., 2016b). However, the electron-transfer pathways in the microorganisms analyzed here are not yet fully understood.

Sulfur metabolism in *Desulfurellaceae* members is quite diverse, as genes encoding for at least three enzymes involved in sulfur reduction are present in the group. Sulfur, sulfide and polysulfide are present in solution in a pH-dependent equilibrium $\left({HS}^{-}+\frac{x-1}{8}S_{8}↔S_{n}^{2-}+H^{+}\right)$. At higher pH values, polysulfide is present as the dominant form, while at low pH values elemental sulfur prevails (Kleinjan et al., 2005).

*Hippea* species genomes possess genes encoding for the membrane bound PSR, an integral membrane protein complex responsible for quinone oxidation coupled to polysulfide reduction, and the cytoplasmic sulfide dehydrogenase (SUDH), reported to catalyze the reduction of polysulfide to hydrogen sulfide with NADPH as the electron donor (Macy et al., 1986; Ma et al., 2000). The domains 4Fe-4S, 4Fe-S Mo-bis of the catalytic subunit and Nfr of the membrane-bound subunit with nine transmembrane helices of the PSR are conserved in all the *Hippea* species. The pH range for growth of *Hippea* species (Miroshnichenko et al., 1999; Flores et al., 2012) supports the hypothesis of sulfur reduction through polysulfide in these microorganisms.

**FIGURE 2:**
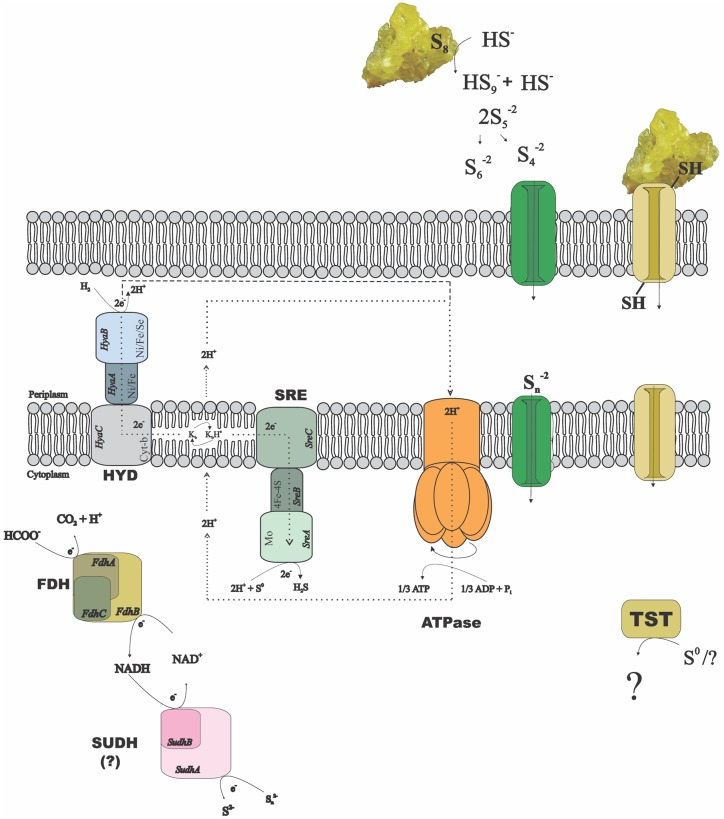
**Possible mechanisms of sulfur/polysulfide respiration in *Desulfurella amilsii*.** During chemolithotrophic growth, hydrogenases (HYD) might transfer electrons to sulfur reductase (SRE) via menaquinones (K) encoded in the genome, and protons to an encoded ATPase, creating a proton motive force. If sulfide dehydrogenase (SUDH) plays a role in sulfur respiration, its cytoplasmic nature hampers the generation of proton motive force by any conventional mechanisms and therefore, it is likely that the membrane-bound hydrogenases pump protons out of the cell to generate a gradient. In case of formate used as electron donor, the intracellular formate dehydrogenase (FDH) encoded might transfer electrons to SUDH, with NAD^+^/NADH as intermediates. Moreover, rhodanese-like proteins (TST) encoded in the genome might have a role in the process, but its performance in sulfur-respiring microorganisms is not yet clearly understood.

The alpha and beta subunits of the SUDH encoded in all genomes of the *Desulfurellaceae* family show domains conserved in all the microorganisms: NAD-binding and iron-sulfur clusters (3Fe-4S and 4Fe-4S) domains in the subunit SudhA and FAD-binding and iron-sulfur cluster 2Fe-2S domains in the subunit SudhB. In *D. acetivorans*, only SUDH-coding genes are present (Desace_0075-0076), which would suggest that polysulfide is the terminal electron acceptor in its respiration process. *D. amilsii* is unique as, in addition to SUDH (DESAMIL20_1852-1853), SRE is encoded (DESAMIL20_1357-1361). A SRE was isolated from the acidophile *Acidianus ambivalens*, and its subunits were partially characterized and compared to their homologous in the PSR isolated from *Wolinella succinogenes* (Laska et al., 2003). SRE is reported to be involved in direct reduction of elemental sulfur, with the electrons being donated by hydrogenase, quinones and cytochrome *c*. SRE also uses NADPH as an electron donor, but at low activity (Laska et al., 2003). The SRE encoded in the *D. amilsii* genome presents, in general, conserved domains for four of its subunits. The membrane anchor subunit (SreC), with nine transmembrane helices, has a PSR domain (**Figure 2**) similar to the one encoded in *A. ambivalens*, which was shown by Laska et al. (2003) to be phylogenetically unrelated to the analogous *W. succinogenes* protein. The catalytic subunit (SreA) contains the conserved molybdopterin domain, predicted to be functional with respect to oxidoreductase activity. The sequence, however, does not present a twin-arginine motif and so, in contrary to the SRE from *A. ambivalens*, it might be cytoplasm oriented. The subunit SreB also presents the 4Fe-4S domain conserved, which has a high degree of sequence similarity to Mo-FeS enzymes of the DMSO reductase family. The subunit SreD in *D. amilsii* does not contain the conserved 4Fe-4S domain; but its function in sulfur respiration is not yet clears (Laska et al., 2003). The *sreE* gene encodes a protein of 209 aa length with similarity to reductase assembly proteins required either for the assembly of the Mo-containing large subunit of DMSO reductase or nitrate reductase (Blasco et al., 1998; Ray et al., 2003).

Since the reduction of elemental sulfur through polysulfide is unlikely at low pH, the SRE encoded in *D. amilsii* might play a role when this microorganism grows in acidic conditions. Moreover, several thiosulfate sulfurtransferases with rhodanese domains are exclusively encoded in *Desulfurella* species.

The enzyme SUDH isolated from *Pyrococcus furiosus* was reported to show SRE activity *in vitro*. However, the expression of its coding-genes also correlated to the carbon source rather than to elemental sulfur/polysulfide, especially when its intracellular concentration is below 1.25 mM (Ma and Adams, 2001). It is likely that this enzyme acts *in vivo* as a ferredoxin:NADPH oxidoreductase (NfnAB). In this case, in *Hippea* species, the sulfur reduction process might be carried out by the PSR, while in *Desulfurella* species the rhodanese-like thiosulfate sulfurtransferases might play an essential role. In **Figure 2**, a metabolic reconstruction of the possible sulfur reduction pathways in *D. amilsii* is depicted.

*Desulfurella amilsii* is able to use thiosulfate as a terminal electron acceptor in a range of pH from 5 to 7, an ability not reported for any of the other analyzed genomes of the members of *Desulfurellaceae*. Although *D. propionica* was also shown *in vivo* to utilize thiosulfate as an electron acceptor, its genome sequence is not yet available. The known pathway of thiosulfate reduction refers to a two-step process, involving the enzymes thiosulfate reductase and the dissimilatory sulfite reductase (Stoffels et al., 2012). The first is reported to be involved in the conversion of thiosulfate into sulfide and sulfite, which can be toxic for most microorganisms. The dissimilatory reductase converts the generated sulfite into sulfide, eliminating the toxicity of sulfite from the medium. In *D. amilsii*, it is likely that thiosulfate respiration occurs via this pathway, as the thiosulfate reductase, the dissimilatory sulfite reductase (DsrAB), the DsrC protein and the subunits DsrM and DsrK of the Dsr MKJOP complex are encoded in the genome. The genome of *D. acetivorans* encodes a thiosulfate reductase and the dissimilatory sulfite reductase, but subunits of the Dsr MKJOP transmembrane complex and the DsrC protein are not encoded. Therefore, the absence of subunits of Dsr MKJOP and DsrC might explain the inability of *D. acetivorans* to respire thiosulfate. **Table 3** summarizes the enzymes involved in sulfur and thiosulfate respiration, with their respective reactions and the orthologs genes.

**Table 3:** Enzymes, reactions and occurrence of orthologous genes involved in elemental sulfur and thiosulfate respiration in *Desulfurellaceae* family.

Enzyme	Reaction	Occurrence of orthologous genes						
		
		Subunits	*Dam*	*Dac*	*Hma*	*Hja*	*Hal*	*Hme*
		PsrA	–	–	0433	1370	0846	0560
Polysulfide reductase	$S_{n}^{2-}\to S^{2-}+S_{n-1}^{2-}$	PsrB	–	–	0434	1371	0847	0561
		PsrC	–	–	0435	1372	0848	0562

2*Sulfide dehydrogenase^∗^	$S_{n}^{2-}\to S^{2-}+S_{n-1}^{2-}$	SudhA	1853	0076	0231	1601	1361	1618
		SudhB	1852	0075	0230	1600	1360	1617

		SreA	1359	–	–	–	–	–
		SreB	1357	–	–	–	–	–
Sulfur reductase	S^0^ → S^2-^	SreC	1358	–	–	–	–	–
		SreD	1360	–	–	–	–	–
		SreE	1361	–	–	–	–	–

		PhsA	9	1254	0433	1171	1675	0227
Thiosulfate reductase	$S_{2}O_{3}^{2-}\to S^{2-}+{SO}_{3}^{2-}$	PhsB	8	1253	–	–	–	–
		PhsC	10	1255	–	1172	1676	0228

2*Sulfite reductase		DsrA	1435	1402	–	–	–	–
		DsrB	1434	1401	–	–	–	–
DsrC	${SO}_{3}^{2-}\to S^{2-}$	DsrC	1431, 2056	–	–	–	–	–
2*Complex Dsr MK		DsrM	1430	–	–	–	–	–
		DsrK	1429	–	–	–	–	–


*Dam, *D. amilsii*; Dac, *D. acetivorans*; Hma, *H. maritima*; Hja, *H. Jasoniae;* Hal, *H. alviniae*; Hme, *H. medeae*. The prefix of the locus tags for the analyzed species are DESAMIL20_ (*D. amilsii*); Desace_ (*D. acetivorans*); Hipma_ (*H. maritima*); EK17DRAFT*_* (*H. jasoniae*); G415DRAFT_ (*H. alviniae*) and D891DRAFT_(*H. medeae*). To avoid repetition of the prefix in the table, all the locus tags are represented only by the specific identifier. ^∗^Possibly functioning as bifurcating/confurcating enzyme.*

*Desulfurella* species grow and produce sulfide and sulfate from sulfur in the absence of an organic electron donor (Florentino et al., 2016a), in a specific redox reaction that undergoes oxidation and reduction, also called disproportionation. Sulfur could be converted into sulfide via a sulfur-reducing enzyme (e.g., SRE/SUDH) and to sulfite by an unidentified enzyme. In general, the sulfite could be oxidized to sulfate by sulfite oxidoreductase (SUOR) or adenosine-5′-phosphosulfate (APS) reductase, with ATP sulfurylase or adenylylsulfate:phosphate adenylyltransferase (APAT) being involved (Finster et al., 1998; Frederiksen and Finster, 2003; Hardisty et al., 2013). Although the enzyme responsible for the conversion of sulfur into sulfite is not known, SUDH/SRE and DSR coding genes were detected in both *Desulfurella* members’ genomes, suggesting that these bacteria might disproportionate elemental sulfur using this pathway. APS reductase was not detected in any species, which supports the inability of this group to use sulfate as electron acceptor or to disproportionate elemental sulfur via the reverse pathway from sulfite to APS and then to sulfate.

Sulfur metabolism in *Desulfurellaceae* family members is quite diverse. The presence of unique proteins in *D. amilsii* might explain its ability to respire elemental sulfur at low pH, where polysulfide is not available. The ability of *D. amilsii* to respire thiosulfate in a two-step process is also unique among the analyzed members of the family. Besides, disproportionation appears as a feature only shared by members of *Desulfurella* genus, and so this genus, with a more versatile metabolism, offers more possibilities for biotechnological application based on sulfidogenesis.

### Other Aspects of *Desulfurellaceae* Members’ Metabolism

**FIGURE 3:**
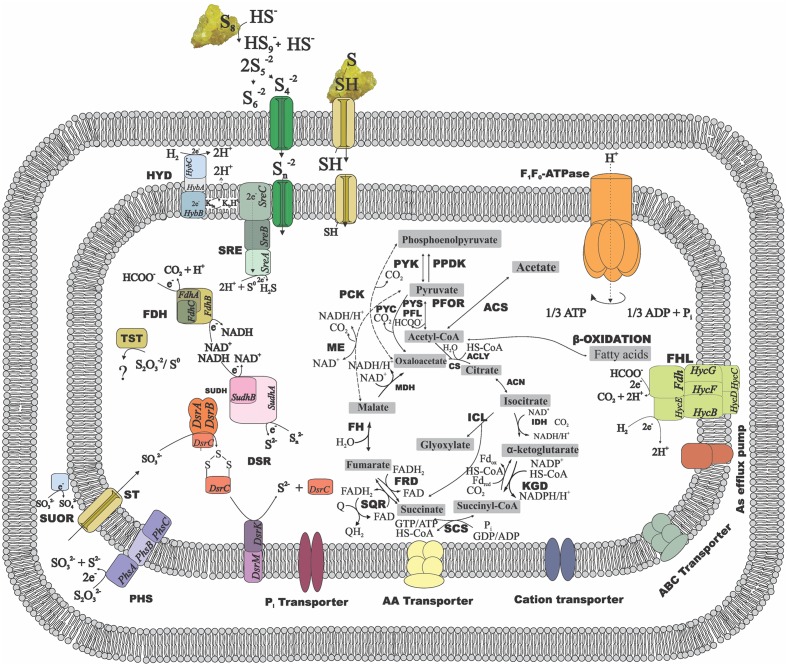
**Metabolic reconstruction of *D. amilsii*.** Acetate, hydrogen or formate are possible electron donors for the given scheme while sulfur or thiosulfate are reflected as electron acceptors. The amino acids, cations and phosphate transporters encoded in the genome and likely involved in resistance to stress conditions are also depicted. ACK, acetate kinase; ACLY, ATP citrate lyase; ACS, acetyl-CoA synthetase; CS, citrate synthase; DSR, Dissimilatory sulfite reductase; FDH, formate dehydrogenase; FH, fumarate hydratase; FHL, formate hydrogen lyase; FRD, fumarate reductase; HYD, hydrogenase; ICL, Isocitrate lyase; IDH, Isocitrate dehydrogenase; KGD, α-ketoglutarate dehydrogenase; MDH, malate dehydrogenase; ME, malic enzymes; MK, menaquinone; PCK, phosphoenolpyruvate carboxinase; PFL, pyruvate:formate lyase; PFOR, pyruvate:ferredoxin oxidoreductase; PHS, thiosulfate reductase; PPDK, pyruvate phosphate dikinase; PTA, phosphotransacetylase; PYC, pyruvate carboxylase; PYK, pyruvate kinase; PYS, Pyruvate synthase; SCS, Succinyl-CoA synthetase; SQR, succinate-coenzyme Q reductase; SRE, sulfur reductase; SUDH, sulfide dehydrogenase; SUOR, sulfite oxidoreductase; TST, thiosulfate sulfurtransferase. The central carbon metabolism in the figure can be extended to all the members of Desulfurellaceae family, as most of the features are conserved among the species. The dashed lines represent exclusive possible conversions for Desulfurella species and the solid lines represent possible conversions common to all members of the studied family.

Enzymes involved in the central carbon metabolism of *Desulfurellaceae* members are listed in **Supplementary Table S2** and the ones involved in energy metabolism and conservation are listed in **Supplementary Table S3**. The general metabolic reconstruction of *D. amilsii* is depicted in **Figure 3**, in which the differential central carbon metabolism for *Desulfurellaceae* members can also be seen. Proteins for complete Embden-Meyerhof-Parnas and oxidative TCA cycle pathways are encoded in all the genomes of the *Desulfurellaceae* members, as well as decarboxylating malate dehydrogenase (ME), which can catalyze the reversible conversion of malate to pyruvate. Although the malate dehydrogenase is present, malate transporters are not encoded in the genome of the analyzed *Desulfurella* genus members, which might explain their inability to use malate as an electron donor for growth.

Besides the conversion of phosphoenolpyruvate to pyruvate via pyruvate kinase (PYK) and the irreversible carboxylation of pyruvate to form oxaloacetate via pyruvate carboxylase (PYC) common for all *Desulfurellaceae* members, *Desulfurella* and *H. jasoniae* genomes also encode the phosphoenolpyruvate carboxylase (PCK). Pyruvate:ferredoxin oxidoreductase (PFOR) and related 2-oxoacid:ferredoxin oxidoreductases are encoded in all the genomes in the group, where pyruvate oxidation is a main intermediate metabolic reaction. Moreover, all the genomes possess the gene encoding pyruvate:formate lyase (PFL), involved in pyruvate metabolism and leading to the production of acetyl-CoA and formate. *D. amilsii* and *D. acetivorans* were shown to ferment pyruvate in laboratorial analyses, but formate could only be used as an electron donor by *D. amilsii* (Florentino et al., 2016a), despite the subunits FdoG, FdoH and FdoI of a FDH being encoded in *D. acetivorans* genome.

All members of the *Desulfurellaceae* family can utilize acetate (Florentino et al., 2016a). The metabolism of acetate starts with its activation to acetyl-CoA, an essential intermediate of various anabolic and catabolic pathways in all forms of life (Ingram-Smith et al., 2006). Acetate activation involves either the enzymes acetyl-CoA synthetase (ACS), acetate kinase (ACK) in combination with phosphate acetyltransferase (PTA), or the enzyme succinyl-CoA: acetate CoA-transferase (SCACT). All *Desulfurellaceae* species have the enzyme ACS encoded in their genome. In *Desulfurella* species, however, acetyl-CoA could also be generated from acetate via acetylphosphate involving ACK and PTA. The genome analysis shows both pathways for acetate oxidation are encoded in *Desulfurella* species. However, experimental studies performed by Schmitz et al. (1990) showed that cell extracts of *D. acetivorans* had high specific activities of ACK (5 U/mg) and PTA (14 U/mg), but no activity of the alternative ACS nor the SCACT. Although Goevert and Conrad (2010) demonstrated acetate activation via ACK and its metabolization via the TCA cycle in *H. maritima*, genes encoding ACK are not found in any *Hippea* members’ genome.

Chemolithotrophic growth of *Desulfurellaceae* members with H_2_ as electron donor and S^0^ as electron acceptor requires at least two enzymes in a short electron transport chain composed by a hydrogenase, an electron carrier, and a sulfur/polysulfide reductase. Only one Ni-Fe type hydrogenase (HybABC), which catalyzes reversible hydrogen production/consumption, is encoded in *Desulfurellaceae* members together with its maturation protein HypABCDEF (**Supplementary Table S3**). The subunit HybB is embedded in the membrane and the subunit HybA possess a tat signal, therefore the hydrogenase is membrane-bound facing periplasm. The hydrogen is converted into protons, creating proton motive force and electrons which are transferred via intramembrane electron carriers, such as the encoded menaquinone, to the membrane bound SRE or PSR, or to the cytoplasmic SUDH.

Although physiological tests revealed some differences among the studied species, the comparative genomic analysis on the general metabolism of *Desulfurellaceae* members does not show great divergence in gene sets involved in chemolithotrophic growth, TCA cycle and pyruvate fermentation. However, the utilization of acetate might have different routes of metabolization by the two analyzed genera.

### Resistance Mechanisms at Low pH

Acidophiles and acidotolerant microorganisms can have a broad range of adaptation mechanisms to thrive at acidic environments, while ensuring higher cytoplasmic pH values than the surrounding environment (Baker-Austin and Dopson, 2007).

It is predicted that *Desulfurella* species can synthesize degradative arginine decarboxylase to consume intracellular protons via the amino acid decarboxylation reaction and, consequently, neutralize the medium. Moreover, the analyzed *Desulfurella* species encode the K^+^-transporting ATPase and a putative regulating histidine kinase, involved in the generation of positive internal membrane potential by influx of potassium ions in order to inhibit the flux of protons in extreme acidophiles (Dopson and Johnson, 2012). ABC phosphate transporters, sodium-coupled antiporters and amino acid antiporters that are pH dependent (Kanjee and Houry, 2013) and related to acid resistance are also encoded in the referred genomes (**Supplementary Table S4**). The genomic components potentially involved in stress response to acidic environments in *Desulfurellaceae* members are listed in **Supplementary Table S4**.

The ability of *Desulfurella* species to thrive at low pH using acetate as an electron donor requires resistance mechanisms. When the pH of the medium is lower than the pKa value of acetic acid (4.75), the weak organic acid prevails in its protonated form, which crosses the cytoplasmic membrane by diffusion. At neutral cytoplasmic pH, the acid dissociates, leading to the release of protons and respective anions, resulting in the acidification of the cytoplasm (Holyoak et al., 1996). *Desulfurella* species genomes encode the ATP-binding cassette transporter (AatA) reported to be involved in acetic acid resistance in acetic acid bacteria (Nakano et al., 2006). This putative ABC transporter contains two ABC motifs in tandem on a single polypeptide, which possibly serves as an exporter of acetic acid, maintaining a low level of intracellular acetic acid concentration (Nakano et al., 2006).

The genes encoded in *Desulfurellaceae* family members possibly involved in resistance to low pH do not vary. However physiological tests showed the ability of *Desulfurella* species to grow at more acidic environments, with *D. amilsii* being able to grow at pH as low as 3 (Florentino et al., 2016a) and *D. acetivorans* at pH 4.3 (Bonch-Osmolovskaya et al., 1990). Different regulation of those genes, or a completely unknown mechanism encoded in those microorganisms, might be key to explain the differences in resistance of high proton concentrations.

### Response to Oxidative Stress

Survival of strict anaerobic microorganisms, such as the members of the *Desulfurellaceae* family, in environments exposed to high redox potential would include antioxidant strategies. Furthermore, the acidotolerant *D. amilsii* was isolated from acidic sediments from the Tinto River which possess zones with very high redox conditions (up to +400mV) and high concentrations of soluble metals, such as copper, iron, and zinc (Florentino et al., 2015). The excess of metals contributes to redox-active metals toxicity, generating reactive oxygen species (ROS) via the slow Fenton and Haber–Weiss reactions. When the oxidation states of the metal ions switches, reactive species, such as hydrogen peroxide (H_2_O_2_) and superoxide (•O^2-^) are activated to the hydroxyl radical (•OH), resulting in a highly reactive form (Flora et al., 2008). Therefore, the presence of genes encoding oxidative stress related enzymes is of great importance for the survival of this species in its original habitat.

Superoxide reductase desulfoferrodoxin is encoded in all *Desulfurellaceae* members’ species, as well as rubredoxin, that can transfer electrons and reduce the superoxide dismutase (**Supplementary Table S5**) (Sheng et al., 2014). Reduction of peroxides is performed by enzymes such as glutathione peroxidase, peroxirredoxin, rubrerythrins, alkylhydroperoxidases and catalases. Rubrerythrin is encoded in all the genomes; in *Desulfurella* species, *H. alviniae* and *H. jasoniae* the rubrerythrin-coding gene is flanked by a peroxiredoxin, while in *H. maritima* and *H. medea* it is flanked by a DNA repair mechanism involved in gene spore photoproduct lyase. Peroxiredoxins and thioredoxins-coding genes are present in all *Desulfurellaceae* genomes studied. Together with rubrerythrin and the ferric uptake regulator (Fur) family, the peroxiredoxins and thioredoxins are well-represented in acidophiles and acidotolerant microorganisms (Cárdenas et al., 2016). The rubrerythrin and the Fur family replace activities of catalase and oxidative stress response regulators in neutrophiles, while peroxiredoxins and thioredoxins remove organic peroxides originated when ROS attack organic molecules (Cárdenas et al., 2012).

Oxidizing agents normally modify the DNA in complex patterns, leading to mutagenic effects. Three different DNA repair pathways are involved in the removal of the oxidized bases in DNA and their mismatches: base excision repair (BER), nucleotide excision repair (NER) and mismatch repair (MMR). The genomes of the *Desulfurella* species encode DNA repair mechanisms, including the protein RecA, the excinuclease UvrABC and the GroEL protein (**Supplementary Table S4**). All bacterial genomes analyzed contained genes for the detection and removal of modified purine and pyrimidine bases (BER pathway), including orthologs of the uracyl-DNA glycosylase gene. The UvrABC repair system for NER pathway, which operates on the removal of bulky lesions from the DNA duplex, was present in the genome of all species. Additionally, genes responsible for the SOS response to DNA damage, RecA/RadA were found in all organisms; LexA, however, is only present in *D. acetivorans.* Genes encoding the Dps protein, endonucleases and the minimal essential complex for mismatched base repair were not detected in any of the analyzed genomes.

Despite the different isolation sources of the *Desulfurellaceae* members and a lack of physiological data from *Hippea* species and *D. acetivorans*, differences in genes encoding resistance to oxidative stress were not detected in the genome, and so regulatory processes might be responsible for them to tackle the harsh conditions.

### Metals Resistance

Several prokaryotes show specific genetic mechanisms of resistance to toxic concentrations of metals in the environment, which include their oxidation or reduction to less toxic valence states, incorporation or precipitation of heavy metals as metal sulfides complexes, and the direct transport of metals out of the membrane (Ji and Silver, 1995). Generally, the mechanisms for uptake of metals can be ATP-independent and driven by chemosmotic gradients across the membrane or is dependent on the energy released from ATP hydrolysis in a substrate-specific manner (Ahemad, 2012).

One of the ATP-based mechanisms proposed for metals resistance in bacteria is the synthesis of polyphosphates via the enzyme polyphosphate kinase, which can interact with metal ions due to its polyanion nature (Pan-Hou et al., 2002). Genes encoding the polyphosphate kinase are present in *Desulfurella* species and in *H. maritima. D. amilsii* was shown to be resistant to relatively high concentrations of copper and nickel (Florentino et al., 2015). The resistance to copper can also be related to the presence of genes encoding the copper-exporting P-type ATPase, present in all species.

*Desulfurella* species and *H. maritima* genomes encode the Co/Zn/Cd efflux system, components of inorganic ion transport and metabolism. *Desulfurella* species and *H. alviniae* encode some cation transporters (**Supplementary Table S6**), that are unspecific and chemiosmotic gradient driven across their cytoplasmic membrane.

Although genes encoding resistance to heavy metals are in all the analyzed species, the isolation source of *D. amilsii* is a metal rich environment, and, as many metals are more soluble at acidic pH, this microorganism is more exposed to the high metal concentrations than the other members of *Desulfurellaceae* family isolated from neutrophilic environments (Bonch-Osmolovskaya et al., 1990; Miroshnichenko et al., 1999; Flores et al., 2012). Besides, as described by Dopson et al. (2014), high concentrations of sulfate are also normally present in acidic environments, which can complex metal cations and lower the concentration of free metals that can enter the microbial cell cytoplasm. Therefore, it is likely that such abiotic factor, in combination with other factors, such as the competition with protons for binding sites, might contribute to the increased tolerance to metals in solution by *D. amilsii* in comparison to its neutrophilic relatives.

## Conclusion

Analysis of available genomes of the *Desulfurellaceae* family provided insight into their members’ energy and carbon metabolism, helping in the elucidation of the genomic diversity in this group of microbes. Comparative genome analysis revealed that the gene content for sulfur respiration differs between genera and within the *Desulfurella* genus. PSR might be the responsible enzyme for indirect sulfur reduction in *Hippea*. SRE is suggested to play a role in sulfur reduction by *D. amilsii*, especially when it grows at low pH. Since the enzyme annotated as SUDH might act as a bifurcating enzyme, respiration of elemental sulfur by *Desulfurella* spp. possibly occurs via other enzymes, such as the encoded rhodanese-like sulfurtransferases. Gene prediction supported by experimental analysis in *Desulfurella* species indicate a more versatile metabolism in this group. Although the ability to grow at extreme acidic environments is only confirmed in *D. amilsii*, great differences in the gene sets involved in the resistance to low pH conditions could not be detected in a comparative genome analysis. Therefore, the regulation of those genes in *D. amilsii*, or a resistance mechanism not yet known, might be responsible for the unique ability of this microorganism to survive in acidic conditions. This is the first report on comparative genomics of sulfur-reducing microorganisms able to grow at different conditions, which might help follow up analyses to broaden the knowledge on this poorly understood group of prokaryotes. Further studies need to be performed to address remaining questions about the active pathways and how environmental conditions interfere with them.

## Author Contributions

AF: Drafting the manuscript; acquisition and analysis of the data; AF and IS-A: Giving substantial contributions to the conception or design of the work; interpretation of data for the article; AF, IS-A, and AS: Agreement to be accountable for all aspects of the work; ensuring that questions related to the accuracy or integrity of any part of the work was properly investigated; IS-A and AS: Revising the manuscript critically for important intellectual content and final approval of the version to be published.

## Conflict of Interest Statement

The authors declare that the research was conducted in the absence of any commercial or financial relationships that could be construed as a potential conflict of interest.

## Footnotes

1 http://www.cbs.dtu.dk/services/TMHMM/

2 http://wlab.ethz.ch/protter/start/

3 https://img.jgi.doe.gov/

## The prefix of the locus tags for the analyzed species are

*D. amilsii*: DESAMIL20_
*D. acetivorans*: Desace_
*H. maritima*: Hipma_
*H. jasoniae*: EK17DRAFT
*H. alviniae*: G415DRAFT_
*H. medeae*: D891DRAFT_
