# Supplementary material for: Genome Sequence of Desulfurella amilsii Strain TR1 and Comparative Genomics of Desulfurellaceae Family
Source: Front Microbiol. 2017 Feb 20;8:222. doi: 10.3389/fmicb.2017.00222 (PMC5317093; doi:10.3389/fmicb.2017.00222)
Supplement: Supplementary file 1 [file Table_1.docx]

Table S1 – Number of coding sequences assigned to COG functions for *Desulfurella amilsii.*

| **COG category** | **Number of CDS** | **Percentage** |
| --- | --- | --- |
| Amino acid transport and metabolism | 134 | 8.27 |
| Carbohydrate transport and metabolism | 63 | 3.89 |
| Cell cycle control, cell division, chromosome portioning | 23 | 1.42 |
| Cell motility | 71 | 4.38 |
| Cell wall/ membrane/ envelope biogenesis | 124 | 7.65 |
| Coenzyme transport and metabolism | 118 | 7.28 |
| Defense mechanisms | 26 | 1.6 |
| Energy production and conversion | 159 | 9.81 |
| Extracellular structures | 17 | 1.05 |
| Function unknown | 52 | 3.21 |
| General function predicted only | 109 | 6.72 |
| Inorganic transport and metabolism | 91 | 5.61 |
| Intracellular trafficking, secretion and vesicular transport | 32 | 1.97 |
| Lipid transport and metabolism | 91 | 5.37 |
| Mobilome: prophages, transposons | 12 | 0.74 |
| Nucleotide transport and metabolism | 55 | 3.39 |
| Post-transcriptional modification, protein turnover, chaperones | 72 | 4.44 |
| Replication, recombination and repair | 72 | 4.44 |
| Secondary metabolites biosynthesis, transport and catabolism | 17 | 1.05 |
| Signal transduction mechanisms | 69 | 4.26 |
| Transcription | 58 | 3.58 |
| Translation, ribosomal structure and biogenesis | 160 | 9.87 |
| No function | 680 | 31.84 |
