# Supplementary material for: Genome Sequence of Desulfurella amilsii Strain TR1 and Comparative Genomics of Desulfurellaceae Family
Source: Front Microbiol. 2017 Feb 20;8:222. doi: 10.3389/fmicb.2017.00222 (PMC5317093; doi:10.3389/fmicb.2017.00222)
Supplement: Supplementary file 2 [file Table_2.docx]

Table S2 – Enzymes involved in the central carbon metabolism of *Desulfurellaceae* members. Dam - *D. amilsii*, Dac – *D. acetivorans*, Hma - *H. maritima*, Hja – *H. jasoniae*, Hal – *H. alviniae*, Hme - *H. medeae*.

|  | **Dam** | **Dac** | | **Hma** | **Hja** | **Hal** | **Hme** |
| --- | --- | --- | --- | --- | --- | --- | --- |
|  |  | | **Embden-Meyerhof-Parnas Pathway** | | | | |
| Phosphoglycerate kinase | 553 | 0175 | | 0158 | 0687 | 1447 | 1655 |
| Glyceraldehyde-3-phosphate dehydrogenase | 554 | 0176 | | 0157 | 0686 | 1448 | 1654 |
| Phosphoglycerate mutase | 558, 730, 106 | 0180, 0837, 1097, 1209 | | 0199, 0839 | 0013, 1208 | 0355, 1774 | 0916, 1647, 1655 |
| Fructose-bisphosphate aldolase | 731, 1935 | 0029, 0836, 1097 | | 1172 | 0083 | 0784 | 1211 |
| Pyruvate kinase | 732 | 0835 | | 0462 | - | 1377 | 1337 |
| phosphopyruvate hydratase | 154, 1024 | 1049 | | 0808 | 1157 | 0689 | 0886 |
| 6-phosphofructokinase | 1865 | 1829 | | 0795 | 1144 | 0702 | 0873 |
| Glucose-6-phosphate isomerase | 1936 | 0030 | | 1170 | 0081 | 0782 | 1209 |
| Fructose-1,6-bisphosphatase | 26 | 1193 | | 0344 | 0779 | 0381 | 0519 |
| Triose phosphate isomerase | 553 | 0174 | | 0159 | 0688 | 1446 | 1656 |
|  |  | | **Pyruvate metabolism** | | | | |
| Malate dehydrogenase | 1542, 1998 | 0393, 0423, 1793 | | 0134 | 1703 | 1462 | 0580 |
| Malic enzyme | 1512 | 0423 | | 0099 | 1753 | 1514 | 1738 |
| Pyruvate kinase | 732 | 0835 | | 0462 | - | 1377 | 1337 |
| Pyruvate carboxylase | 537 | 0154 | | 0131 | 1482 | 0581 | 1706 |
| Phosphoenolpyruvate carboxylase | 942 | 0682 | | - | - | 1002 | - |
| Pyruvate phosphate dikinase | 1215 | 1460 | | 0076 | 1539 | 1730 | 1762 |
| Pyruvate synthase | 1627-1628, 1974 | 0299-0300,  1025, 1810 | | 0589-0591, 0797-0798 | 0875-0876, 0729-0732 | 0357-0358, 1017-1018, 1146-1147 | 0699-0700, 1021-1022 |
| Pyruvate-ferredoxin/flavodoxin oxidoreductase | 1627-1628, 1974 | 0299-0300,  1025, 1810 | | 0589-0591, 0797-0798 | 0729-0732, 0875-0876 | 0357-0358, 1017-1018, 1146-1147 | 0699-0700, 1021-1022 |
| Acetate kinase | **1989** | **1802** | | - | - | - | - |
| Phosphotransacetylase | 1988 | 1803 | | 0099 | 1738 | 1753 | 1514 |
| Pyruvate: formate lyase | 541, 1072, 1223, 1707 | 0159, 0553, 0806, 1468, 1619 | | 1145 | 0186 | 1268 | 1308 |
|  |  | | **Acetate oxidation** | | | | |
| Acetyl-CoA synthetase | 135, 719, 1370, 1743, | 1068, 1651 | | 0577, 1234 | 0292-0293, 0718, 1325, 1422, | 0277, 1400 | 0040-0041, 0197-0198, 0406, 1321 |
| Acetate kinase | **1989** | **1802** | | - | - | - | - |
| Phosphate acetyl transferase | 222 | 0978 | | 1059 | 1138 | 0338 | 0534 |
| CO Dehydrogenase | - | **1218, 1220, 1221** | | - | - | - | - |
| Citrate synthase | 790, 1597, 1709 | 0509, 1333, 1621, 1814 | | - | - | - | - |
|  |  | **TCA Cycle** | | | | | |
| Malate/lactate dehydrogenase | 1542, 1998 | 0393, 0423, 1793 | | 0134 | 1703 | 1462 | 0580 |
| Succinyl-CoA synthetase | 830-831, 908-909 | 0507-0508, 0719-0720, 0758-0759, 1310-1311 | | 1409-1410 | 0453-0454 | 1788-1789 | 0727-0728 |
| Fumarate hydratase class I | 1540-1541 | 0394-0395 | | 0135-0136 | 1701-1702 | 1460-1461 | 0578-0579 |
| Fumarate hydratase class II | **1637** | **0290** | | - | - | - | - |
| Citrate synthase | 790, 1597, 1709 | 0509, 1333, 1621, 1814 | | 0545 | 0687 | 1197 | 0241 |
| Isocitrate/isopropylmalate dehydrogenase | 247, 865 | 0953 | | 0452 | 0580 | 0805 | 1330 |
| Succinate dehydrogenase/fumarate reductase | 1536-1537 | 0396-0399 | | 0139-0140 | 1697-1698 | 1456-1457 | 0574-0575 |
| Pyruvate/2-oxoglutarate dehydrogenase complex E2 and E3 | 623, 1860, 2035 | 1234, 1824 | | 0389 | 1131 | 0411 | 1766 |
| Aconitase | 969, 1840 | 0088, 0656 | | 0218 | 1630 | 1373 | 1613 |

The prefix of the locus tags for the analysed species are: DESAMIL20_ (*D. amilsii*); Desace_ (*D. acetivorans*); Hipma_ (*H. maritima*); EK17DRAFT*_* (*H. jasoniae*); G415DRAFT_ (*H. alviniae*) and D891DRAFT_ (*H. medeae*). To avoid repetition of the prefix in the table, all the locus tags are represented only by the specific identifier
