# Supplementary material for: Genome Sequence of Desulfurella amilsii Strain TR1 and Comparative Genomics of Desulfurellaceae Family
Source: Front Microbiol. 2017 Feb 20;8:222. doi: 10.3389/fmicb.2017.00222 (PMC5317093; doi:10.3389/fmicb.2017.00222)
Supplement: Supplementary file 3 [file Table_3.docx]

Table S3 – Enzymes involved in the energy conservation and metabolism of *Desulfurellaceae* members. Dam - *D. amilsii*, Dac – *D. acetivorans*, Hma - *H. maritima*, Hja – *H. jasoniae*, Hal – *H. alviniae*, Hme - *H. medeae*.

|  | **Dam** | | | **Dac** | | **Hma** | **Hja** | | **Hal** | | **Hme** | |
| --- | --- | --- | --- | --- | --- | --- | --- | --- | --- | --- | --- | --- |
|  |  | **Electron transport chain** | | | | | | | | | | |
| Ni-Fe type hydrogenase HybABC | 502- 504 | | | 0124-0126 | | 1096-1098 | 1668-1670 | | 0120-0122 | | 1196-1198 | |
| Hydrogenase maturation protein (HypABCDEF) | 499-501, 505-508 | | | 0121-0123,0127-0130 | | 1093-1095 | 1665-1667 | | 0127-0129 | | 1193-1195 | |
| Fe_S hydrogenase | 510, 1789 | | | 0132, 1217 | | 1488 | 1519 | | - | | 0330 | |
| formate hydrogenlyase | 1015-1020 | | | 0604-0609 | | 1312-1317 | 1467-1472 | | 1381-1386 | | 1492-1497 | |
| Menaquinone | 264 | | | 0937 | | 1576 | 0558 | | 1708 | | 0215 | |
| Polysulfide reductase | - | | | - | | **0433-0435** | **1370-1372** | | **0846-0848** | | **0560-0562** | |
| Sulfide dehydrogenase | 1852- 1853 | | | 0075-0076 | | 0230-0231 | 1600-1601 | | 1360-1361 | | 1617-1618 | |
| Sulfur reductase | **1357- 1361** | | | - | | - | - | | - | | - | |
| Thiosulfate reductase | 8-10 | | | 1253-1255 | | 0433 | 1170-1172 | | 1674-1676 | | 0226-0228 | |
| Rhodanese-like thiosulfate sulfurtransferase | **270, 1100, 1419, 1987, 2007** | | | **0521, 0931, 1491, 1783, 1804** | | **-** | - | | - | | - | |
| Sulfite oxidoreductase | **1907** | | | **1876** | | - | - | | - | | - | |
| Dissimilatory sulfite reductase DsrAB | **1434-1435** | | | **1401-1402** | | - | - | | - | | - | |
| Dissimilatory reductase DsrC | **1431** | | | **-** | | - | - | | - | | - | |
| Complex Dsr MK | **1429-1430** | | | **-** | | - | - | | - | | - | |
|  |  | **CO_2_ fixation** | | | | | | | | | | |
| CO dehydrogenase | - | | | **1220-1221** | | - | - | | - | | - | |
| Acetyl-CoA synthase | 1743, 135 | | | 1068, 1651 | | 0577, 1234 | 0277, 1400 | | 0197-0198 | | 0718, 1422 | |
| Fumarate reductase | 1536-1537 | | | 0396-0399 | | 0140 | 0574 | | 1456 | | 1697 | |
| Ferredoxin-dependent 2-oxoglutarate synthase | 1790 | | | 1699-1703 | | 1489 | 1518 | | 0081 | | 0329 | |
| ATP-Citrate lyase | 1597-1598 | | | 0509 | | - | - | | - | | - | |
| 2-oxoglutarate carboxylase / pyruvate carboxylase | 537 | | 0154 | | 0131 | | | 0581 | | 1482 | | 1706 |
| Isocitrate dehydrogenase | 247, 865 | | | 0953, 1028 | | 0452 | 1330 | | 0805 | | 0580 | |
| Pyruvate synthase | 1627-1628, 1974 | | | 0299-0300,  1025, 1810 | | 0589, 0797-0798 | 0357,1018, 1146-1147 | | 0700, 1023-1024 | | 0729-0731, 0875 | |
| Phosphoenolpyruvate carboxylase | 942 | | | 0682 | | - | - | | 1002 | | - | |
| Acetyl/propionyl-CoA carboxylases | 800, 1110, 1521 | | | 0414-0415, 0511, 1322-1323, | | 0703, 0822 | 0364, 1189 | | 0963 | | 0798 | |

The prefix of the locus tags for the analysed species are: DESAMIL20_ (*D. amilsii*); Desace_ (*D. acetivorans*); Hipma_ (*H. maritima*); EK17DRAFT*_* (*H. jasoniae*); G415DRAFT_ (*H. alviniae*) and D891DRAFT_ (*H. medeae*). To avoid repetition of the prefix in the table, all the locus tags are represented only by the specific identifier
