# Supplementary material for: Genome Sequence of Desulfurella amilsii Strain TR1 and Comparative Genomics of Desulfurellaceae Family
Source: Front Microbiol. 2017 Feb 20;8:222. doi: 10.3389/fmicb.2017.00222 (PMC5317093; doi:10.3389/fmicb.2017.00222)
Supplement: Supplementary file 4 [file Table_4.docx]

Table S4 – Enzymes potentially involved in the resistance to acidic environments in *Desulfurellaceae* members. Dam - *D. amilsii*, Dac – *D. acetivorans*, Hma - *H. maritima*, Hja – *H. jasoniae*, Hal – *H. alviniae*, Hme - *H. medeae*.

|  | **Dam** | | **Dac** | | | | **Hma** | **Hja** | | | **Hal** | | | **Hme** | | | |
| --- | --- | --- | --- | --- | --- | --- | --- | --- | --- | --- | --- | --- | --- | --- | --- | --- | --- |
|  |  | **DNA repair** | | | | | | | | | | | | | | |  |
| Protein RecA | 136 | | 1067 | | | | 0248 | 1583 | | | 1343 | | | 1599 | | | |
| Excinuclease ABC | 757 | | 0811 | | | | 1579 | 0253 | | | 1154 | | | 0699 | | | |
| GroEL | 1280 | | 1539 | | | | 1268 | 0228 | | | 1106 | | | 1450 | | | |
|  |  | **Decarboxylases** | | | | | | | | | | | | | | |  |
| Arginine decarboxylase | 90 | | | 1123 | 0369, 0390 | | | | | 1641 | | | 0210 | | | 1318 | |
|  |  | **Symporters/Antiporters** | | | | | | | | | | | | | | |  |
| Sodium coupled symporters | 786 | | 0782 | | | | 0364, 0382 | - | | | - | | | - | | | |
| Sodium coupled antiporter | 1647, 1684, 1515 | 0280, 0420, 1269 | | | | 0365, 0382 | | |  | | |  | | |  | | |
| Amino acid antiporter | 256-257, 411, 565-566, 572-573, 766, 1489-1490, 1465, 1533, 1783, 2003, | | 0138, 0187-0188, 0194-0195, 0213, 0402, 0444-0445, 0555, 0802, 0909, 0943-0944, 1132, 1228, 1438, 1522 | | | | 0013, 0045, 0097, 0741, 1007, 1619 | 0273- 0274, 0445, 0560, 0655, 0656 0673-0674 0823- 0824, 0898, 1199, 1543 | | | 0352, 0475-0476, 1484-1487, 1628-1631, 1714- 1717 | | | 0080 - 0081, 0200-0201, 0354, 0720,  1085-1086, 1311, 1710- 1711 | | | |
|  |  | **Phosphate transport** | | | | | | | | | | | | | | |  |
| ABC transporter | 206, 1152 | | 0996 | | | | 0887-0890 | 937 | | | 0607, 0925 | | | 967 | | | |
|  |  | **Membrane potential** | | | | | | | | | | | | | | |  |
| Histidine kinase | 1383 | | 0069, 0580, 0993, 1174, 1347, 1467, 1486, 1544 | | | | 0349-0352, 1470 | 0367, 0784, 0940, 1537 | | | 0102, 0960, 1083 | | | 0348, 0429, 0795, 0964 | | | |

The prefix of the locus tags for the analysed species are: DESAMIL20_ (*D. amilsii*); Desace_ (*D. acetivorans*); Hipma_ (*H. maritima*); EK17DRAFT*_* (*H. jasoniae*); G415DRAFT_ (*H. alviniae*) and D891DRAFT_ (*H. medeae*). To avoid repetition of the prefix in the table, all the locus tags are represented only by the specific identifier
