# Supplementary material for: Genome Sequence of Desulfurella amilsii Strain TR1 and Comparative Genomics of Desulfurellaceae Family
Source: Front Microbiol. 2017 Feb 20;8:222. doi: 10.3389/fmicb.2017.00222 (PMC5317093; doi:10.3389/fmicb.2017.00222)
Supplement: Supplementary file 5 [file Table_5.docx]

Table S5 – Enzymes involved in the response to oxidative stress in *Desulfurellaceae* members. Dam - *D. amilsii*, Dac – *D. acetivorans*, Hma - *H. maritima*, Hja – *H. jasoniae*, Hal – *H. alviniae*, Hme - *H. medeae*.

|  | **Dam** | | **Dac** | | **Hma** | **Hja** | | **Hal** | | **Hme** | |
| --- | --- | --- | --- | --- | --- | --- | --- | --- | --- | --- | --- |
|  |  | **Hydrogen peroxide stress** | | | | | | | | | |
| Peroxide stress regulator | 548, 1785 | | 0169, 1698 | | 1578 | 0553 | | 1703 | | 0213 | |
| Alkylhydroperoxide | 547, 965 | | 0708, 1020, 1233 | | 0417 | - | | - | | - | |
| Rubrerythrins | 1608 | | 0320, 0651, 0654 | | 1117 | 0088 | | 0854 | | 1216 | |
| Peroxiredoxin | 547, 930, 965 | | 0168, 0652, 0697, 1118 | | 0750 | 1107, 1303 | | 0715, 0853 | | 0545, 0829 | |
| Thioredoxin | 1131 | | 0490 | | 0617, 1036 | 0401, 1367 | | 0431, 0991 | | 0202, 0762, 1111 | |
| Fur family | 80, 548, 1785 | | 0169, 1698, 1131 | | 1578 | 0553 | | 1703 | | 0213 | |
|  |  | **Superoxide stress** | | | | | | | | | |
| Redox-sensitive transcriptional activator SoxR | **1086** | | **0161, 1585** | | - | - | | - | | - | |
| Rubredoxin | 1603 | | 0325 | | 0415 | 1391 | | 0856 | | 0543 | |
| Superoxide dismutase desulfoferrodoxin | 1606 | | 0322 | | 0419 | 1395 | | 0857 | | 0547 | |
|  |  | **Redox and oxygen sensors** | | | | | | | | | |
| Methyl-accepting chemotaxis protein | 283,  386,  491,  532,  1098, 1379,  1382,  1594 | | 0113,  0150,  0336,  0523,  0920-0921,  1483 | | 0599, 0628-0629, 0758, 0907, 1171, 1274, 1236 | 0054, 0082, 0222, 0413, 0901, 1031, 1057, 1111,  1312 | | 0166, 0602, 0610, 0783, 0989, 1009,  1070, 1169, 1112 | | 0119, 0539, 0641, 0744, 0989, 1041, 1182, 1210, 1424, 1456, 1489, 1490 | |
| Flagellar motor rotation proteins | 1791-1792 | 1705 | | 0575 | | | 0279 | | 0195 | | 0716 |
| Quinol oxidase - cytochrome bd type | 1439 | | 1408 | | 1558 | 1443 | | 1405 | | 0154 | |
|  |  | **DNA repair** | | | | | | | | | |
| UvrABC system | 757 | | 0811 | | 0557 | 0253 | | 1154 | | 0699 | |
| Uracil- DNA glycosylase | 1848 | | 0080 | | 0225 | 1606 | | 1366 | | 1623 | |
| Protein RecA | 136 | | 1067 | | 0248 | 1583 | | 1343 | | 1599 | |
| LexA | - | | 1741 | | - | - | | - | | - | |

The prefix of the locus tags for the analysed species are: DESAMIL20_ (*D. amilsii*); Desace_ (*D. acetivorans*); Hipma_ (*H. maritima*); EK17DRAFT*_* (*H. jasoniae*); G415DRAFT_ (*H. alviniae*) and D891DRAFT_ (*H. medeae*). To avoid repetition of the prefix in the table, all the locus tags are represented only by the specific identifier
