# Supplementary material for: Genome Sequence of Desulfurella amilsii Strain TR1 and Comparative Genomics of Desulfurellaceae Family
Source: Front Microbiol. 2017 Feb 20;8:222. doi: 10.3389/fmicb.2017.00222 (PMC5317093; doi:10.3389/fmicb.2017.00222)
Supplement: Supplementary file 6 [file Table_6.docx]

Table S6 – Enzymes involved in the resistance to metals toxicity in *Desulfurellaceae* members. Dam - *D. amilsii*, Dac – *D. acetivorans*, Hma - *H. maritima*, Hja – *H. jasoniae*, Hal – *H. alviniae*, Hme - *H. medeae*.

|  | **Dam** | | **Dac** | | **Hma** | **Hja** | | | **Hal** | | | **Hme** | | | |
| --- | --- | --- | --- | --- | --- | --- | --- | --- | --- | --- | --- | --- | --- | --- | --- |
|  |  | **ATP-based** | | | | | | | | | | | | |  |
| Polyphosphate kinase | 882 | | 0746, 1836, 1840 | | 0825 | | | 0472 | | | 1084 | | |  |  |
| Copper-exporting P-type ATPase | 1050 | | 1817 | | 0745 | 0377 | | | 1148, 1792 | | | 1705 | | | |
|  |  | **Non-ATP based** | | | | | | | | | | | | |  |
| Cation transporters | 49, 597 | | 0185, 0214, 0705, 1171 | | 1423, 1426-1427 | 0179, 1365, 1676 | | | 1178 | | | 0441 | | | |
| ABC-type zinc and iron transporters | 215-217 | | 0983-0985 | | 1038-1040 | 1361-1363 | | | 0427-0429 | | | 1113-1115 | | | |
| Zinc-chromate transporters | **1196** | | - | | - | - | | | - | | | - | | | |
|  |  | **Enzymatic reduction** | | | | | | | | | | | | |  |
| Uptake of selenite (DedA protein) | 6, 266 | | 935, 1257 | | 0495, 1447 | 1055, 1662 | | | 0833, 1066 | | | 0433, 0634 | | | |
| Arsenic efflux pump | 1318 | 1269 | | - | | | - | | | - | | | - | | |
| Arsenic resistance operon | **1348-1349** | - | | - | | | - | | | - | | | - | | |

The prefix of the locus tags for the analysed species are: DESAMIL20_ (*D. amilsii*); Desace_ (*D. acetivorans*); Hipma_ (*H. maritima*); EK17DRAFT*_* (*H. jasoniae*); G415DRAFT_ (*H. alviniae*) and D891DRAFT_ (*H. medeae*). To avoid repetition of the prefix in the table, all the locus tags are represented only by the specific identifier
