# Supplementary material for: Genome Sequence of Desulfurella amilsii Strain TR1 and Comparative Genomics of Desulfurellaceae Family
Source: Front Microbiol. 2017 Feb 20;8:222. doi: 10.3389/fmicb.2017.00222 (PMC5317093; doi:10.3389/fmicb.2017.00222)
Supplement: Supplementary file 7 [file Table_7.docx]

Table S7 – Distance-matrix of 16S rRNA sequences of the members of *Desulfurellaceae* family. The distance percentages were estimated using the Jukes-Cantor correction algorithm. Dam - *D. amilsii*, Dac – *D. acetivorans*, Dmu – *D. multipotens*, Hma - *H. maritima*, Hja – *H. jasoniae*, Hal – *H. alviniae*, Hme - *H. medeae*.

|  | ***Dam*** | ***Dac*** | ***Dmu*** | ***Hal*** | | ***Hja*** | | ***Hma*** | ***Hme*** |
| --- | --- | --- | --- | --- | --- | --- | --- | --- | --- |
| ***Dam*** | - | 97.7 | 97.7 | 84.5 | 86.7 | | 87.6 | | 87.0 |
| ***Dac*** |  | - | **99.9** | 85.7 | 87.0 | | 87.8 | | 88.0 |
| ***Dmu*** |  |  | - | 85.5 | 87.1 | | 88.2 | | 88.1 |
| ***Hal*** |  |  |  | - | 94.8 | | 92.1 | | 94.7 |
| ***Hja*** |  |  |  |  | - | | 95.4 | | 96.6 |
| ***Hma*** |  |  |  |  |  | | - | | 97.0 |
| ***Hme*** |  |  |  |  |  | |  | | - |
