# Supplementary material for: Genome Sequence of Desulfurella amilsii Strain TR1 and Comparative Genomics of Desulfurellaceae Family
Source: Front Microbiol. 2017 Feb 20;8:222. doi: 10.3389/fmicb.2017.00222 (PMC5317093; doi:10.3389/fmicb.2017.00222)
Supplement: Supplementary file 8 [file Table_8.docx]

Table S8 – Unique genes encoded in *D. amilsii*.

| **Locus Tag** | **Gene Name** |
| --- | --- |
| 7 | hypothetical protein |
| 11 | hypothetical protein |
| 12 | hypothetical protein |
| 13 | hypothetical protein |
| 14 | voltage-gated potassium channel |
| 15 | hypothetical protein |
| 24 | hypothetical protein |
| 37 | hypothetical protein |
| 76 | hypothetical protein |
| 78 | hypothetical protein |
| 81 | hypothetical protein |
| 89 | hypothetical protein |
| 95 | acetoin utilization protein AcuB |
| 156 | hypothetical protein |
| 234 | hypothetical protein |
| 274 | hypothetical protein |
| 275 | hypothetical protein |
| 276 | hypothetical protein |
| 284 | Uncharacterized conserved protein YbjQ, UPF0145 family |
| 285 | Tetratricopeptide repeat-containing protein |
| 286 | TraX protein |
| 287 | hypothetical protein |
| 289 | hypothetical protein |
| 290 | hypothetical protein |
| 292 | DNA polymerase III sliding clamp (beta) subunit, PCNA homolog |
| 293 | hypothetical protein |
| 294 | hypothetical protein |
| 295 | dUTP pyrophosphatase |
| 296 | hypothetical protein |
| 298 | DNA topoisomerase-3 |
| 299 | hypothetical protein |
| 300 | hypothetical protein |
| 301 | hypothetical protein |
| 302 | hypothetical protein |
| 304 | hypothetical protein |
| 305 | hypothetical protein |
| 306 | AAA-like domain-containing protein |
| 307 | hypothetical protein |
| 308 | conjugation TrbI-like protein |
| 309 | hypothetical protein |
| 310 | hypothetical protein |
| 311 | type IV conjugative transfer system protein TraL |
| 312 | hypothetical protein |
| 313 | hypothetical protein |
| 314 | Type II secretory pathway, component PulF |
| 315 | prepilin-type N-terminal cleavage/methylation domain-containing protein |
| 317 | hypothetical protein |
| 318 | prepilin-type N-terminal cleavage/methylation domain-containing protein |
| 319 | prepilin-type N-terminal cleavage/methylation domain-containing protein |
| 320 | hypothetical protein |
| 321 | hypothetical protein |
| 322 | hypothetical protein |
| 323 | transposase, IS605 OrfB family, central region |
| 324 | hypothetical protein |
| 325 | hypothetical protein |
| 326 | hypothetical protein |
| 330 | hypothetical protein |
| 331 | transposase, IS605 OrfB family, central region |
| 333 | hypothetical protein |
| 384 | hypothetical protein |
| 397 | hypothetical protein |
| 398 | hypothetical protein |
| 399 | hypothetical protein |
| 403 | hypothetical protein |
| 404 | transposase, IS605 OrfB family, central region |
| 406 | hypothetical protein |
| 408 | transposase, IS605 OrfB family, central region |
| 409 | hypothetical protein |
| 410 | hypothetical protein |
| 460 | hypothetical protein |
| 485 | Major Facilitator Superfamily protein |
| 489 | transposase, IS605 OrfB family, central region |
| 517 | Cupin domain-containing protein |
| 519 | Uncharacterised ArCR, COG2043 |
| 534 | hypothetical protein |
| 593 | cobalt-zinc-cadmium efflux system protein |
| 594 | hypothetical protein |
| 595 | hypothetical protein |
| 612 | hypothetical protein |
| 614 | hypothetical protein |
| 615 | Uncharacterized protein, UPF0261 family |
| 616 | Predicted TIM-barrel enzyme |
| 617 | pyruvate dehydrogenase E1 component alpha subunit |
| 618 | pyruvate dehydrogenase E1 component beta subunit |
| 619 | hypothetical protein |
| 620 | pyruvate dehydrogenase E1 component alpha subunit |
| 621 | pyruvate dehydrogenase E1 component beta subunit |
| 622 | methylmalonyl-CoA epimerase |
| 630 | hypothetical protein |
| 631 | hypothetical protein |
| 633 | hypothetical protein |
| 634 | hypothetical protein |
| 635 | PH domain-containing protein |
| 638 | hypothetical protein |
| 639 | Antirestriction protein ArdC |
| 640 | hypothetical protein |
| 641 | hypothetical protein |
| 642 | hypothetical protein |
| 643 | hypothetical protein |
| 644 | hypothetical protein |
| 645 | hypothetical protein |
| 646 | hypothetical protein |
| 647 | hypothetical protein |
| 648 | hypothetical protein |
| 649 | hypothetical protein |
| 651 | Transglycosylase SLT domain-containing protein |
| 652 | hypothetical protein |
| 654 | hypothetical protein |
| 656 | UvrD/REP helicase N-terminal domain-containing protein |
| 657 | hypothetical protein |
| 658 | Transglycosylase SLT domain-containing protein |
| 659 | hypothetical protein |
| 660 | Helicase conserved C-terminal domain-containing protein |
| 661 | hypothetical protein |
| 662 | hypothetical protein |
| 663 | hypothetical protein |
| 664 | hypothetical protein |
| 665 | hypothetical protein |
| 666 | hypothetical protein |
| 667 | Toprim-like |
| 669 | hypothetical protein |
| 671 | hypothetical protein |
| 672 | hypothetical protein |
| 673 | hypothetical protein |
| 674 | hypothetical protein |
| 675 | hypothetical protein |
| 676 | hypothetical protein |
| 678 | hypothetical protein |
| 679 | protein of unknown function DUF87 |
| 682 | hypothetical protein |
| 683 | hypothetical protein |
| 684 | TraU protein |
| 685 | hypothetical protein |
| 686 | hypothetical protein |
| 687 | hypothetical protein |
| 688 | TraG-like protein, N-terminal region |
| 689 | hypothetical protein |
| 690 | hypothetical protein |
| 691 | hypothetical protein |
| 692 | hypothetical protein |
| 695 | hypothetical protein |
| 696 | ERF superfamily protein |
| 697 | hypothetical protein |
| 698 | CRISPR/Cas system-associated exonuclease Cas4, RecB family |
| 699 | hypothetical protein |
| 701 | hypothetical protein |
| 703 | transposase, IS605 OrfB family, central region |
| 705 | hypothetical protein |
| 706 | hypothetical protein |
| 707 | hypothetical protein |
| 718 | benzoyl-CoA reductase, subunit C |
| 720 | hypothetical protein |
| 806 | hypothetical protein |
| 839 | FlgN protein |
| 842 | hypothetical protein |
| 843 | hypothetical protein |
| 844 | hypothetical protein |
| 845 | hypothetical protein |
| 849 | Na+/H+-dicarboxylate symporter |
| 850 | aspartate racemase |
| 852 | transposase |
| 855 | (2R)-sulfolactate sulfo-lyase subunit alpha |
| 856 | (2R)-sulfolactate sulfo-lyase subunit beta |
| 857 | Tripartite-type tricarboxylate transporter, receptor component TctC |
| 858 | putative tricarboxylic transport membrane protein |
| 859 | Tripartite tricarboxylate transporter TctB family protein |
| 860 | L-alanine-DL-glutamate epimerase |
| 861 | hypothetical protein |
| 867 | hypothetical protein |
| 868 | hypothetical protein |
| 870 | hypothetical protein |
| 871 | Flavin reductase like domain-containing protein |
| 874 | hypothetical protein |
| 876 | Uncharacterized protein YuzE |
| 877 | protein of unknown function (DUF4258) |
| 879 | Antitoxin Phd_YefM, type II toxin-antitoxin system |
| 880 | hypothetical protein |
| 938 | hypothetical protein |
| 976 | hypothetical protein |
| 1010 | hypothetical protein |
| 1023 | Right handed beta helix region |
| 1025 | MutS domain V |
| 1026 | MutS domain V |
| 1053 | hypothetical protein |
| 1056 | hypothetical protein |
| 1059 | hypothetical protein |
| 1083 | hypothetical protein |
| 1094 | hypothetical protein |
| 1115 | hypothetical protein |
| 1121 | hypothetical protein |
| 1139 | hypothetical protein |
| 1164 | hypothetical protein |
| 1185 | PLD-like domain-containing protein |
| 1186 | hypothetical protein |
| 1188 | L,D-transpeptidase catalytic domain |
| 1189 | hypothetical protein |
| 1190 | Predicted arabinose efflux permease, MFS family |
| 1191 | Uncharacterized protein YcsI, UPF0317 family |
| 1192 | Metallo-beta-lactamase superfamily protein |
| 1193 | UPF0271 protein |
| 1194 | sensor histidine kinase inhibitor, KipI family |
| 1195 | biotin-dependent carboxylase uncharacterized domain-containing protein |
| 1196 | chromate transporter |
| 1199 | hypothetical protein |
| 1204 | hypothetical protein |
| 1206 | hypothetical protein |
| 1207 | Uncharacterized protein, contains HEPN domain, UPF0332 family |
| 1208 | hypothetical protein |
| 1209 | TIGR04255 family protein |
| 1211 | Restriction endonuclease |
| 1213 | hypothetical protein |
| 1214 | hypothetical protein |
| 1217 | hypothetical protein |
| 1228 | hypothetical protein |
| 1234 | hypothetical protein |
| 1247 | hypothetical protein |
| 1248 | DNA helicase-2 / ATP-dependent DNA helicase PcrA |
| 1251 | type II restriction enzyme |
| 1345 | hypothetical protein |
| 1348 | transcriptional regulator, ArsR family |
| 1349 | arsenite transporter, ACR3 family |
| 1350 | 4Fe-4S binding domain-containing protein |
| 1351 | hypothetical protein |
| 1352 | Carboxymuconolactone decarboxylase family protein |
| 1353 | RND family efflux transporter, MFP subunit |
| 1355 | phosphonate transport system substrate-binding protein |
| 1356 | histidine kinase |
| 1357 | two component transcriptional regulator, LuxR family |
| 1358 | Sulfur reductase subunit C |
| 1359 | Sulfur reductase subunit A |
| 1360 | Sulfur reductase subunit D |
| 1361 | Sulfur reductase subunit E |
| 1362 | hypothetical protein |
| 1366 | hypothetical protein |
| 1389 | hypothetical protein |
| 1404 | hypothetical protein |
| 1406 | hypothetical protein |
| 1471 | hypothetical protein |
| 1472 | protein of unknown function (DUF4917) |
| 1473 | Superfamily I DNA or RNA helicase |
| 1474 | hypothetical protein |
| 1477 | hypothetical protein |
| 1503 | hypothetical protein |
| 1596 | hypothetical protein |
| 1633 | hypothetical protein |
| 1674 | hypothetical protein |
| 1692 | hypothetical protein |
| 1727 | hypothetical protein |
| 1729 | hypothetical protein |
| 1744 | hypothetical protein |
| 1797 | KUP system potassium uptake protein |
| 1828 | hypothetical protein |
| 1892 | hypothetical protein |
| 1925 | UDP-glucose:(heptosyl)LPS alpha-1,3-glucosyltransferase |
| 1952 | T/G mismatch-specific endonuclease |
| 1953 | Z1 domain-containing protein |
| 1954 | NgoFVII restriction endonuclease |
| 1955 | DNA (cytosine-5)-methyltransferase 1 |
| 1959 | Methyltransferase domain-containing protein |
| 1960 | Putative flippase GtrA (transmembrane translocase of bactoprenol-linked glucose) |
| 1961 | hypothetical protein |
| 1979 | hypothetical protein |
| 1980 | hypothetical protein |
| 1981 | hypothetical protein |
| 1982 | hypothetical protein |
| 2010 | hypothetical protein |
| 2025 | hypothetical protein |
| 2026 | hypothetical protein |
| 2033 | hypothetical protein |
| 2034 | hypothetical protein |
| 2035 | dihydrolipoamide dehydrogenase |
| 2038 | hypothetical protein |
| 2044 | hypothetical protein |
| 2045 | two component transcriptional regulator, LuxR family |
| 2046 | Signal transduction histidine kinase |
| 2047 | hypothetical protein |
| 2048 | Cytochrome c553 |
| 2049 | hypothetical protein |
| 2050 | O-acetylhomoserine sulfhydrylase |
| 2051 | cyclic pyranopterin phosphate synthase |
| 2052 | homoserine O-acetyltransferase |
| 2053 | methionine biosynthesis protein MetW |
| 2054 | sulfide:quinone oxidoreductase |

The prefix of the locus tags for *D. amilsii* is DESAMIL20_.To avoid repetition of the prefix in the table, all the locus tags are represented only by the specific identifier
